# Macacine Herpesvirus 1 Antibody Prevalence and DNA Shedding among Invasive Rhesus Macaques, Silver Springs State Park, Florida, USA

**DOI:** 10.3201/eid2402.171439

**Published:** 2018-02

**Authors:** Samantha M. Wisely, Katherine A. Sayler, C. Jane Anderson, Carisa L. Boyce, Amy R. Klegarth, Steve A. Johnson

**Affiliations:** University of Florida, Gainesville, Florida, USA (S.M. Wisely, K.A. Sayler, C.J. Anderson, C.L. Boyce, S.A. Johnson);; University of Washington, Seattle, Washington, USA (A.R. Klegarth)

**Keywords:** invasive species, nonhuman primates, noninvasive sampling, zoonotic disease, viruses, zoonoses, United States, herpes B, herpes B virus, rhesus macaques, *Macaca mulatta*, macacine herpesvirus 1, McHV-1, Silver Springs State Park, Florida, virus shedding, shedding, antibody prevalence, free-ranging, saliva

## Abstract

We compiled records on macacine herpesvirus 1 (McHV-1) seroprevalence and, during 2015–2016, collected saliva and fecal samples from the free-ranging rhesus macaques of Silver Springs State Park, a popular public park in central Florida, USA, to determine viral DNA shedding and perform sequencing. Phylogenetic analysis of the US5 and US5-US6 intragenic sequence from free-ranging and laboratory McHV-1 variants did not reveal genomic differences. In animals captured during 2000–2012, average annual seroprevalence was 25% ± 9 (mean ± SD). We found 4%–14% (95% CI 2%–29%) of macaques passively sampled during the fall 2015 mating season shed McHV-1 DNA orally. We did not observe viral shedding during the spring or summer or from fecal samples. We conclude that these macaques can shed McHV-1, putting humans at risk for exposure to this potentially fatal pathogen. Management plans should be put in place to limit transmission of McHV-1 from these macaques.

Nonhuman primates (NHPs) can harbor multiple zoonotic pathogens owing to their close phylogenetic relationship and frequent interactions with humans and can be successful ecologic invaders (*1*). In the United States, >10 NHP species have been introduced. Of these, the rhesus macaque (*Macaca mulatta*) has successfully established populations in Florida and Puerto Rico (*2**,**3*).

Rhesus macaques are the most frequently used NHP species in biomedical research (*4*). In laboratory settings, rhesus macaques are considered an occupational health threat because they harbor macacine herpesvirus 1 (McHV-1, also known as herpes B virus). Although the infection does not produce clinical illness in macaques, ≈50% of infections cause fatal encephalitis in humans if left untreated (*5*). Transmission has typically occurred via exposure to macaque bodily fluids, including bites and scratches, although an instance of human-to-human transmission has been reported (*6*). Like other alphaherpesviruses, after the initial infection in macaques, McHV-1 becomes latent in the trigeminal ganglia and lumbosacral nerve. During periods of stress or immunosuppression in the macaque, the virus can reactivate into the lytic phase, at which time the virus sheds from oral, nasal, or genital mucosa without signs of clinical illness (*7*). In the laboratory setting, ≈40% of seropositive rhesus macaques had viral DNA in the trigeminal ganglia, indicating these animals were carriers of the virus and capable of reactivation (*8*).

Outside of the laboratory setting, little is known about the risk for transmission or the incidence of human disease resulting from McHV-1 exposure. No human deaths have been reported from contracting McHV-1 from free-ranging macaques, suggesting the risk for transmission from these animals is low (*9*); however, immunologic surveillance, reporting, and diagnostic investigations in humans are lacking. Aside from characterizing the incidence in humans, the components of transmission risk from free-ranging macaques to humans have not been well characterized. Prevalence of McHV-1 antibodies in free-ranging macaque populations are highly variable (e.g., 72% in Puerto Rico, 64% in Nepal, and 5.3% in Indonesia) (*10**–**12*), and only 1 publication reports the prevalence of viral DNA shedding among captively held, wild macaques (long-tailed macaques, *M. fascicularis*; 39%) (*13*). In that study, viral shedding rates of wild macaques were likely inflated because animals were held in captivity for <72 hours, which could have induced a stress response and therefore viral shedding. Comparisons of the genetic composition of viruses circulating in free-ranging and captive macaques are also lacking yet necessary to understand if differences in virulence or pathogenicity are evident. Estimating the frequency of virus shedding and the genetic composition of the virus is a first step toward understanding the public health risk.

In the US state of Florida, the rhesus macaque is an invasive species with a high reproductive capacity, the ability to spread in geographic distribution, and the potential to threaten native fauna with extinction (*14*). Macaques were introduced into Silver Springs State Park in Florida in the 1930s in an effort to increase tourism (*15**,**16*). The population was founded with ≈12 animals from an unknown source in 2 separate introduction events. The population grew to a peak of ≈400 animals in the 1980s and spread into adjacent forests along the Ocklawaha River (*15*). At the beginning of our investigation (2015), ≈175 rhesus macaques lived in Silver Springs State Park (*3*).

McHV-1 antibody production was first detected in this population in 1992 (*17*). Of 29 trapped animals, 12 (41%) were found to be seropositive (*17*). During 1984–2012, ≈1,000 animals were removed intermittently by permit-holding private trappers, but the practice of animal trapping has ended because of public controversy (*3*). As of December 12, 2017, no population management plans were in place.

The purpose of our study was to describe the seroprevalence of McHV-1 by using macaque samples collected by trappers during 2000–2012 in Silver Springs State Park and along the Ocklawaha River in central Florida. We further aimed to determine if McHV-1 viral DNA was being shed by free-ranging rhesus macaques passively sampled in Silver Springs State Park during 2015–2016 and to compare antigenic regions of the virus among genotypes recovered from laboratory and free-ranging animals.

## Materials and Methods

### Serology

During 2000–2012, private trappers collected blood samples from the femoral vein of 317 rhesus macaques along the Silver and Ocklawaha Rivers in Marion County, Florida, across 5 trapping sessions. The trappers sent samples to a commercial laboratory (BioReliance Corporation, Rockville, MD, USA) to evaluate serologic status using an McHV-1 antigen-based ELISA. We considered samples with an optical density >40 positive for McHV-1 antibodies. We reported age, as determined by body mass and dentition, for 213 animals. For the 213 animals for which serologic and age data were available, we used a logistic regression model to determine if age was related to serostatus using R version 3.3.3 (https://www.r-project.org/).

### Saliva and Fecal Sample Collection

Human visitors to the park are most likely to be exposed to McHV-1 through contact with saliva from macaque bites and scratches (*18*) or from contact with virus shed through urine and feces (*5*); therefore, during 2015–2016, we collected saliva and fecal samples from 2 social groups of rhesus macaques in the park that were most habituated to humans (*19*). We collected samples during 3 seasons considered stressful for animals: in the fall of 2015 during the breeding season, in the spring of 2016 during gestation, and in the summer of 2016 during lactation. Stress has been shown to reactivate the McHV-1 virus and promote virus shedding in rhesus macaques (*20*), but whether seasonal stress in free-ranging animals is sufficient to induce virus shedding was not known. We collected saliva samples (n = 121) by giving 1.25-inch oral swabs (Salimetrics, State College, PA, USA) soaked in sucrose solution to the macaques. We monitored the animals as they obtained, chewed, and discarded swabs. When possible, we collected the swabs immediately after they were discarded and recorded the age and sex of the animal that handled the swab. Because multiple animals in a group were present when field personnel distributed swabs, determining which macaque deposited saliva on the oral swab was not always possible. Therefore, we used observational data to estimate a minimum and maximum number of animals sampled. We defined the minimum number of animals sampled as the number of unique animals we observed chewing on cotton swabs presented to the social group within a season. We defined the maximum number of animals sampled as the total number of cotton swabs chewed on by macaques minus the number of samples that we knew were duplicates from a single animal. For both serologic and viral shedding data, we estimated CIs for single group prevalence using Wilson approximation of the exact limits for a binomial distribution (*21*).

We collected fresh feces (n = 21) in the field using sterile plastic spatulas during the spring and summer of 2016. In addition, we collected fecal samples from 2 macaques during necropsies conducted by Florida Fish and Wildlife Conservation Commission veterinarians. One sample came from a 1-year-old male animal found dead in the park in March 2016 and the other from a subadult female animal struck by a car on a highway adjacent to Silver Springs State Park in July 2016. We applied a virus-inactivating buffer containing guanidine (Buffer VXL; QIAGEN, Valencia, CA, USA) to saliva swab samples and fecal samples and placed them on ice packs during collection; immediately after finishing collection, we stored samples at −80°C. In addition to these samples, 10 soil samples were collected in the field as negative controls; these samples were handled, stored, and extracted in parallel to the oral and fecal specimens obtained from live animals.

### DNA Purification and Genetic Analyses

We extracted genomic DNA from 121 saliva, 23 fecal, and 10 soil samples with commercial QIAGEN kits (QIAamp cador Pathogen Mini Kit for saliva and soil, QIAamp Stool Mini Kit for feces) using the manufacturer’s protocols. To detect McHV-1 viral DNA, we performed real-time PCR (rPCR) targeting a 124-bp fragment of the glycoprotein G (gG) gene, shown to be sensitive and specific to McHV-1, as previously described (*22*) using primers gGBV-323F and gGBV-446R and hydrolysis probe gGBV-403T (Table 1). We used the rPCR protocol from the published assay with the following modifications: the PCR reaction contained VetMAX-Plus qPCR Master Mix (Applied Biosystems, Foster City, CA, USA) with 1 µL VetMAX Xeno Internal Positive Control-VIC Assay (Applied Biosystems) and 2 µL of DNA template in a 25-µL reaction. We performed PCR amplification and detection on an ABI Prism 7500 Fast machine (Applied Biosystems) with the following cycling conditions: 10 min at 95°C followed by 40 cycles of 95°C for 15 s and 60°C for 60 s. We confirmed positive samples by performing reactions in triplicate. For samples that tested positive by rPCR assay, we later re-extracted DNA without the internal control DNA to avoid incorporation of control nucleic acids in conventional PCR products and then sequenced by Sanger methods. We quantified re-extracted DNA using a NanoDrop 1000 Spectrophotometer (ThermoFisher Scientific, Waltham, MA, USA). We included molecular grade water on all plates as a negative control.

**Table 1:** PCR oligonucleotide primers and probe used in the detection of McHV-1 viral DNA in samples from rhesus macaques and soil, Silver Springs State Park, Florida, USA, 2000–2012*

PCR target	Reference	Sequence, 5′ → 3′
gGBV-323F	(*21*)	TGGCCTACTACCGCGTGG
gGBV-446R	(*21*)	TGGTACGTGTGGGAGTCGCG
gGBV-403T	(*21*)	(6-FAM)CCGCCCTCTCCGAGCACGTG(BHQ-1)
DFA	(*22*)	GAYTTYGCNAGYYTNTAYCC
ILK	(*22*)	TCCTGGACAAGCAGCARNYSGCNMTNAA
KG1	(*22*)	GTCTTGCTCACCAGNTCNACNCCYTT
TGV	(*22*)	TGTAACTCGGTGTAYGGNTTYACNGGNGT
IYG	(*22*)	CACAGAGTCCGTRTCNCCRTADAT
HB2A	(*8*)	CCGCGCTCGCCACGGACACCA
HB2B	(*8*)	ATCGCGCGCCGGACCGATCGT
AS9	(*23*)	TC[A/T]CCCGGGCTAGACTT[T/C][A/C]TCTTCCTGCTCAG
AS2	(*23*)	ATGGCGGCCAGGGTCAGCGCGCAGAGG
AS8	(*23*)	CTCTGCGCGCTGACCCTGGCCGCCATGG
AS7	(*23*)	CACGTCGGGGGG[G/A]TCCGTCTTCTGCTCC

*BHQ-1, black hole quencher 1; 6-FAM, 6-fluorescein amidite; gG, glycoprotein G; McHV-1, macacine herpesvirus 1.

To confirm the presence of and genetically characterize McHV-1 DNA in positive samples, we amplified and sequenced a segment of the polymerase gene conserved among viruses of the *Herpesviridae* family using primers DFA, ILK, GK1, TGV, and IYG as previously reported (Table 1) (*23*). In addition, we targeted gene US5, which encodes glycoprotein J (gJ), and part of the intergenic region between US5 and US6 using primers HB2A and HB2B as previously reported (Table 1) (*8*). The 50-µL PCR reaction contained LA Taq (Takara, Shiga, Japan). The thermogenic profile was modified to 94°C for 1 min, followed by 35 cycles of 98°C for 15 s, 60°C for 30 s, and 72°C for 60 s, with a final extension at 72°C for 10 min. We visualized PCR products on 2% agarose gels stained with RedView (Genecopoeia, Rockville, MD, USA). To verify the sequence of US5, we used AS9, AS2, AS8, and AS7 (*24*) to primer walk the region between the 3′ end of the US4 gene and the US6 gene. We purified amplicons of the appropriate size from each conventional PCR assay using the QIAquick PCR Purification Kit (QIAGEN) and submitted them to the Interdisciplinary Core for Biotechnology Research at the University of Florida (Gainseville, Florida, USA) for bidirectional sequencing by Sanger methods. We analyzed chromatographs using BioEdit (http://www.mbio.ncsu.edu/BioEdit/bioedit.html) or FinchTV (http://en.bio-soft.net/dna/FinchTV.html) and used MEGA version 7 (http://www.megasoftware.net/) to trim unreadable ends and form consensus sequences between forward and reverse primers. We performed a phylogenetic analysis of the amino acid sequence in MEGA. We aligned using the MUSCLE algorithm and constructed phylogenetic trees using the maximum-likelihood method and the Poisson model with 1,000 bootstrap replicates (*25*).

## Results

### Serology

Of the 317 rhesus macaque blood samples, 84 were seropositive for McHV-1 (Table 2), for an annual average (± SD) of 25% (± 9%). As predicted for a directly transmitted pathogen and as previously reported for McHV-1 (*26**–**28*), exposure to the pathogen, as determined by positive serostatus, increased with age (*x*) (logit[*p*(*x*)] = −4.44 + 1.07*x*; odds ratio 2.9 [95% CI 1.74–4.83]; p = 0.0001).

**Table 2:** Seroprevalence of McHV-1 in rhesus macaques, Silver Springs State Park, Florida, USA, 2000–2012*

Year sample collected and animal age, y	No. samples	No. seropositive	% Seropositive (95% CI)
2000			
<1	2	0	0 (0–66)
1	20	0	0 (0–16)
2	9	1	11 (2–43)
3	11	2	18 (5–48)
4	11	7	64 (35–85)
>5	28	19	68 (49–82)
2001			
<1	3	0	0 (0–56)
1	22	0	0 (0–15)
2	5	1	20 (4–62)
3	1	0	0 (0–80)
4	2	1	50 (9–90)
>5	18	10	56 (34–75)
Unknown	32	18	56 (39–72)
2009			
Unknown	51	9	18 (10–30)
2010			
Unknown	51	8	16 (8–28)
2012			
1	34	0	0 (0–10)
2	10	2	20 (6–51)
3	4	3	75 (30–95)
4	0	0	NA
>5	3	3	100 (44–100)

*The annual average seroprevalence was 25% ± 9% (mean ± SD). McHV-1, macacine herpesvirus 1; NA, not applicable.

### Virus Shedding

Three (2.5%) of 121 oral swab specimens tested positive in triplicate for McHV-1 DNA by rPCR (Table 3). In addition, all conventional PCR assays of these samples were positive, and sequences were verified by using Sanger methods (GenBank accession nos. MG266705–7). BLAST (https://blast.ncbi.nlm.nih.gov) analysis of the polymerase gene fragment yielded 100% identity to McHV-1. The complete US5 gene sequence was identical in 2 of the 3 positive samples, sharing 100% identity with McHV-1 strain M12-0 (GenBank accession no. KY628985), isolated from a captive bonnet macaque (*M. radiata*), and strain 16293 (GenBank accession no. KY628972; Figure), isolated from a captive rhesus macaque (*29*). The US5 sequence generated from the third positive swab specimen differed at a single nucleotide, which resulted in an amino acid change from aspartic acid to glycine at position 28 (nt 84) of the coding region of the gJ gene. The sequence generated from this specimen (GenBank accession no. MG266707) shared 100% identity with 9 previously sequenced isolates collected from captive NHPs (rhesus macaques and a Japanese macaque [*M. fuscata*]) and an isolate originating from primate kidney cells (Figure). The coding sequences obtained from all 3 samples were highly similar (98.3%–100%) to sequences generated from laboratory strains of McHV-1 originating from captive rhesus macaques (*29*).

**Table 3:** Shedding of McHV-1 in rhesus macaque saliva samples collected using 121 oral swabs and quantified on the basis of observational data and rPCR positivity for McHV-1 DNA, by social group, by season, Silver Springs State Park, Florida, USA, 2015–2016*

Season	Group 1				Group 2		
	Minimum no. sampled	Maximum no. sampled	No. rPCR positive		Minimum no. sampled	Maximum no. sampled	No. rPCR positive
Fall 2015, breeding season	11	18	0		10	29	3†
Spring 2016, gestation period	3	13	0		2	9	0
Summer 2016, lactation period	2	11	0		2	26	0

*McHV-1, macacine herpesvirus 1; rPCR, real-time PCR. †These 3 saliva samples represent 2 or 3 unique macaques. We estimated a conservative prevalence of 7% (95% CI 2%–22%) assuming the 3 positive oral swabs came from 2 animals and all 29 animals were sampled. Taking the least conservative approach, we estimated a prevalence of 30% (95% CI 11%–60%) if the 3 swabs came from 3 unique animals and only 10 animals were sampled.

**Figure:**
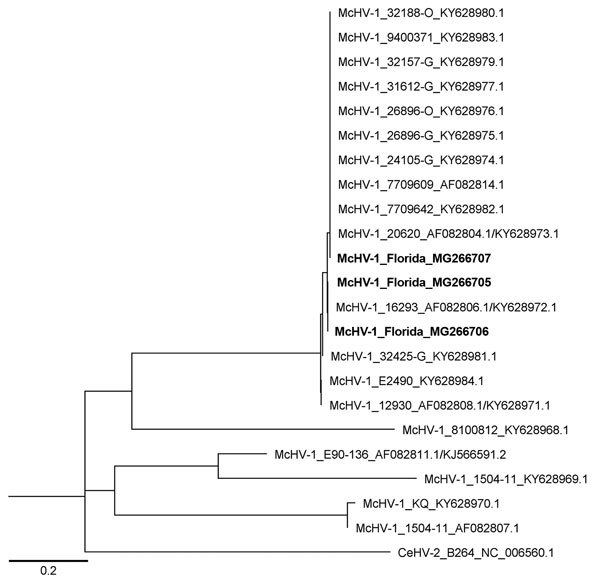
Maximum-likelihood tree of McHV-1 constructed by using highly variable 375-bp fragment of US5 and intergenic region between US5 and US6 genes. The genotypes recovered from free-ranging rhesus macaques (*Macaca mulatta*) in Florida (bold) separate into 2 clades that include laboratory strains of McHV-1. GenBank accession numbers are provided. Scale bar indicates number of base pair changes per nucleotide. McHV-1, macacine herpesvirus 1.

We detected McHV-1 DNA in macaque samples only during the fall (breeding season) of 2015 and not during the spring (gestation period) or summer (lactation period) of 2016 (Table 3). On the basis of the estimated minimum and maximum number of animals sampled, shedding occurred in a minimum of 7% (95% CI 2%–22%) and a maximum of 30% (95% CI 11%–60%) of the animals in 1 of 2 social groups sampled during this time period. For both of the sampled groups combined, the percentage of animals shedding in the fall was a minimum of 4% (95% CI 1%–14%) and a maximum of 14% (95% CI 5%–35%) of all animals sampled in the fall. We did not find evidence of viral shedding in feces (n = 23), and all environmental controls (n = 10) tested negative for McHV-1.

## Discussion

We found evidence that invasive rhesus macaques in Silver Springs State Park shed McHV-1 DNA. Serologic results from the animals trapped during 2000–2012 were consistent with other serologic findings for free-ranging macaques. Although the average (± SD) annual seroprevalence was 25% (± 9%), this number is likely an underestimate of the actual seroprevalence because trappers in later years targeted primarily younger animals, rather than a set of animals reflective of the demographic composition of the population. Among the animals for which we had age class data, younger animals had a lower seroprevalence of McHV-1 (0% for <1-year-olds) than older animals (mean ± SD 57% ± 10% for 4-year-olds and 75% ± 23% for >5-year-olds). These data suggest that a substantial portion of the population are likely carriers for McHV-1 and capable of reactivation and viral shedding.

In our study, we found 2 genotypes of herpes B virus that varied by a single amino acid change. Both of these genotypes have been found in laboratory populations, suggesting that >2 different laboratory-like strains circulate in the park population of macaques. A second observation from our study was that viral shedding appeared to have temporal variation. We only observed animals shedding virus during the breeding season in the fall. This time of year is particularly stressful because male–male aggression is high; consequently, the mortality (*30*) and dispersal (*31*) rates of male animals are highest during this time of year. Indeed, 1 of the positive samples that came from an identified animal was from a subadult male. A better understanding of the demographic and temporal trends of viral shedding by macaques in the park is needed to quantify the risk of exposure to McHV-1 among humans.

During 1977–1984, the Florida Fish and Wildlife Commission documented at least 23 bites that occurred during human-macaque incidents near or in Silver Springs State Park and resulted in human injury (*17*), but this rate is likely an underestimate of the number of bites or scratches that occur each year. Boaters on the Silver River frequently feed the macaques (0.68 provisioning events/h) at close distances (<2 m) (*32*), which creates a high potential for human-macaque encounters and appears to alter the movement of the macaques. Animals spend more time in close proximity to the Silver River on weekends, when boater traffic is highest (*32*), than on weekdays (*19*). This human behavior probably exacerbates the public health threat of the rhesus macaque population to park visitors because incidents of negative human-macaque interactions increase when macaques are fed by humans (*33*). In 2016 and 2017, public areas of the park were closed on multiple occasions because of the aggressive behavior of these macaque groups (personal observation by C.J.A.).

Although the potential for transmission of virus to humans clearly exists in Silver Springs State Park, no human infections or deaths caused by McHV-1 from free-ranging animals have been reported, despite frequent human-macaque interactions among many macaque populations worldwide. All documented cases of human contraction of and death from McHV-1 have been associated with captive animals within laboratory settings. Multiple explanations exist for these paradoxical observations, and none are mutually exclusive. First, multiple strains of the virus might circulate, and the strains present in laboratories might be more pathogenic to humans than those circulating in free-ranging macaques. Second, free-ranging macaques might shed the virus less frequently than captive macaques, which decreases the transmission potential. Third, McHV-1 infection in humans transmitted from free-ranging macaques might be misdiagnosed or underreported (*18*).

The first explanation for the apparent difference in macaque-human transmission rates does not appear to be true in this system. The gJ gene sequences found in viruses in macaques in Silver Springs State Park were identical to isolates originating from laboratories (Figure), suggesting that strain divergence has not occurred. This gene is partially responsible for virulence by inhibiting apoptosis and regulating cellular processes (*34*) and could be a potential gene controlling pathogenicity. However, information from 1 gene enables limited inference; whole-genome sequencing of virus isolates from laboratory and free-ranging animals is needed to understand the role of pathogenicity and the mechanisms responsible for differences among host populations.

The second explanation is that virus is shed more frequently in laboratory animals, thus exposing humans in this setting more frequently, but data to test this hypothesis are sparse. In a study of 1 laboratory population, 9 (75%) of 12 antibody-positive rhesus macaques tested positive via rPCR for viral shedding in mucosa (*35*). In another study, free-ranging long-tailed macaques were captured, transported, and held in captivity for <72 hours before buccal sampling occurred; 154 (39%) of 392 animals tested positive for viral DNA via oropharyngeal and urogenital sampling (*13*). In animals we tested by rPCR, the viral shedding prevalence of 4%–14% was lower than that reported for either the captively bred population (*35*) or the free-ranging animals that were held in captivity (*13*). These observations support the hypothesis of higher shedding rates in captive animals due to the stress associated with high densities, disruption of social structure, and being in an unfamiliar environment (*36*). However, not all laboratory exposures, including those from needle sticks and mucosal splashes, have resulted in seroconversion or any disease manifestation (*37*), nor have bites by pet macaques been linked to any herpes B cases in humans (*38*). More studies are needed to definitively determine the risk for herpes B virus transmission and to more precisely conclude that laboratory animals shed more frequently or have more pathogenic strains.

The third explanation for increased apparent transmission of McHV-1 to humans in a laboratory setting is provided by a difference in probability of detection among human populations (laboratory workers versus the general public). The etiology of encephalitis, which is the manifestation of McHV-1 infection in humans, is undetermined for 30%–60% of encephalitis cases in the United States because of the rapid and nonspecific onset of disease; the transient nature of the viremia; and the lack of rapid, specific diagnostic tests (*39*). Diagnosis of viral encephalitis is likely even lower in much of Asia, where human-macaque interactions are common and surveillance is lacking. Thus, it is plausible that McHV-1 transmission to humans from free-ranging macaques has been underdetected and underreported. Because robust surveillance is lacking, conducting comparative studies on the viruses from laboratory and free-ranging animals is imperative to better determine genomic, epigenetic, and epidemiologic factors associated with transmission and pathogenicity and the role host stress plays in disease outcomes in humans.

Given the current information available, we must consider the presence of the population of invasive rhesus macaques in Florida to be a public health concern. We have shown evidence of viral shedding of McHV-1 in free-ranging macaques at the popular public park, Silver Springs State Park. Although shedding rates appear lower than in captive settings, the potential for human-macaque contact in this park is high. Thoroughly characterizing and comparing the whole genomes of McHV-1 isolates is crucial to deciphering the relationship between pathogenic laboratory strains and strains circulating in the free-ranging macaques in Silver Springs State Park. As of December 12, 2017, no evidence of human transmission from free-ranging macaques exists. However, this pathogen should be considered a low-incidence, high-consequence risk, and adequate public health measures should be taken (*40*).
